# A Study of Psychological Features in Patients With Type 2 Diabetes Mellitus

**DOI:** 10.7759/cureus.70782

**Published:** 2024-10-03

**Authors:** Boris G Tilov, Pavel Stanchev, Maria Orbetsova, Elena Becheva, Petar Antonov, Atanas S Ivanov

**Affiliations:** 1 Department of Psychology, Medical University of Plovdiv, Plovdiv, BGR; 2 Department of Endocrinology, Medical University of Plovdiv, Plovdiv, BGR; 3 Department of Urology and General Medicine, Medical University of Plovdiv, Plovdiv, BGR

**Keywords:** mental adaptability, type 2 diabetes, glycated hemoglobin, aggressiveness, perceived stress, emotional regulation

## Abstract

Aim: There is a lack of multidisciplinary studies examining the link between psychological factors and glycemic control in individuals with chronic illnesses. The aim of this study is to investigate the relationship between psychological factors such as resilience, perceived stress, emotional regulation, aggressiveness, and glycated hemoglobin (HbA1c) levels in patients with type 2 diabetes. Additionally, the study seeks to determine the predictive value of perceived stress and resilience on HbA1c levels and to explore the role of anger expression and emotion regulation strategies in glycemic control, comparing diabetic patients to healthy controls.

Materials and methods: The study was conducted between November 2021 and November 2023 at the Clinic of Endocrinology and Metabolic Diseases at the St. George University Hospital, Bulgaria, and the Department of Science and Research at the Medical University of Plovdiv, Bulgaria. Of these 84 individuals were diagnosed with type 2 diabetes, divided into two groups of 42 individuals each, who had poor and fair glycated hemoglobin. The third group was a healthy control consisting of 42 individuals in the same age group who had no established chronic diseases.

Results: When comparing the study groups on HbA1c and individual psychological characteristics, there were statistically significant differences in resilience, perceived stress, emotion regulation suppression, and anger expression. When comparing the mean values of mental resilience with glycated hemoglobin levels, we find that there are statistically significantly higher mean values between the poor HbA1c control and the healthy group. From the regression analysis, we conclude that the psychological characteristics positively associated with perceived stress (β=0.502; p<0.001) and inversely associated with mental resilience (β=-0.359; p<0.001) are the most predictive. Less influential was the straight correlation with emotion regulation-expressive inhibition (β=0.226; p<0.05), the positive correlation with anger (β=0.170; p<0.001), and general aggressiveness (β=0.151; p<0.05).

Conclusion: From the present study, we note that glycated hemoglobin level is strongly influenced by two psychological predictors, namely subjective perception of stressful situations and resilience level.

## Introduction

The opinion about the social significance of diabetes mellitus as a worldwide disease is widely accepted, in which 90% of patients suffer from type 2. In addition to the clear somatic causes of occurrence, there is a real need for multidisciplinary studies to increase the range of predictors. To this aspect, we can relate the connection of psychological factors to the somatic basis, which has an influence both on the process of the occurrence of the disease itself and on its course - personality traits, cognitive subjective processes, emotional changes, and behavioral characteristics [1,2].

The studies looking for a link between psychosomatic disorders [3], including chronically ill people with type 2 diabetes mellitus (T2DM), and psychological characteristics [4,5] are significantly few in number [6].

Some research in the field focuses on personality-specific individual psychological characteristics in diabetic patients, demonstrating a difference in typology relating to emotional and behavioral characteristics, which highlight the presence of predominant hostility, anger, and ambition [7,8] and emotional instability, social isolation, negative emotions, neuroticism, and psychoticism [9-11].

According to other studies in the field, the psychological features that are found in patients who have endocrine disorders, and in particular T2DM, are emotional instability, guilt, and depression [12-14].

Part of the effective change to improve the quality of life of these patients may be the variety of psychological therapies [15-17], increasing the psychological resilience and proper emotional self-regulation of these patients [16-18], leading to the prevention of perceived stress, anxiety, and depressive symptoms [19-21].

Psychological characteristics that are on the positive spectrum, such as mental resilience, have been insufficiently studied [17,22-24]. Some studies have found a link between positive psychological characteristics and physical health and adaptation in patients with diabetes mellitus [17,25,26]. Psychological resilience can support both the prevention of T2DM and lead to improved quality of life and physical health in this diagnosis [17,27,28]. A long-term follow-up study in the field shows that low mental resilience is a modeling factor for glycosylated hemoglobin (HbA1c) levels, behavioral health self-control, and overall well-being in patients with diabetes [18,29].

Studies in the field noting a link between diabetes and coping strategies [19,30] have found that those who are maladaptive, emotion-focused, and associated with avoidance and disengagement are directly related and presuppose both poor metabolic control, problematic psychological adaptation, and increased anxiety [20,31].

Another important psychological characteristic that is well-studied is stress. In health science, stress is associated with a variety of physical outcomes and diseases, including diabetes mellitus and other chronic diseases [23].

## Materials and methods

The study was conducted at the Clinic of Endocrinology and Metabolic Diseases at the St. George University Hospital, Bulgaria, and the Department of Science and Research at the Medical University of Plovdiv, Bulgaria. The study was conducted in the period from November 2021 to November 2023. At the stage of the study, the subjects did not suffer from COVID-19 and did not report any maladaptive behavior or negative experiences of being in a pandemic situation. The study is cross-sectional. The survey was conducted with the help of a physician, endocrinologist, and clinical psychologist. Additionally, the study is voluntary, anonymous, and absolutely independent of the treatment. The participants in the survey were acquainted with its aim and gave their informed consent.

The initial study included 208 participants who met the inclusion criteria, among whom were patients diagnosed with diabetes mellitus. After applying specific exclusion criteria, the total number of participants was reduced to 126, with the following groups excluded: patients diagnosed with type 1 diabetes, patients with decompensated organ failure or severe clinical conditions, patients with mental illnesses, and patients diagnosed with oncological diseases within the last five years. Participant selection was done using a simple randomization method where each participant had an equal probability of being selected for each group. A random number generator was used to allocate participants to the groups, ensuring that subjective influence was eliminated in the selection process. Randomization was performed using Microsoft Excel (function: RAND), which generates random numbers corresponding to eligible participants.

HbA1c levels were measured by high-performance liquid chromatography (HPLC), which is recognized as the gold standard for accurate HbA1c determination. In this study, we used an automated HPLC analyzer offering high sensitivity and specificity in HbA1c measurement. The analyzer was calibrated in accordance with the National Glycohemoglobin Standardization Program (NGSP) and International Federation of Clinical Chemistry (IFCC) guidelines, ensuring that the results obtained adhered to international standards for data quality and comparability. Measurements were taken at the time of study participant enrollment to ensure consistency across groups.

For the needs of the examination, a control group of persons without established chronic disease was determined. The three groups (HbA1c>7.5%, HbA1c<7.5%, and healthy controls) were compared with the methods for mental resilience (CD-RISC), perceived stress (PSS), emotional regulation (ERQ) (with two subscales: reappraisal (REAS) and suppression (SUP)), and aggression (reported on four scales: physical aggression (FA), verbal aggression (VA), anger (ANG), hostility (HOS), and general aggression (GA)).

In order to confirm or reject the hypothesis of the study, a hierarchical stepwise regression analysis was performed. Based on the model, we will determine some of the factors that presuppose the levels of glycosylated hemoglobin. The analysis was conducted step by step as we accepted as dependent variables the distribution of HbA1c, while predictors are resultant signs (resilience, perceived stress, emotional regulation-reappraisal, emotional regulation-suppression, anger, general aggression, factorial signs (sex, age, weight, physical activity, alcohol intake, smoking, coffee intake, presence or absence of a sleep problem).

Questionnaires

The time for completing the questionnaires was uniform and fixed for all participants, a total of one astronomical hour. Each participant was instructed to answer freely, as quickly as possible, reducing the time for social desirability. The questionnaire battery has a separate social desirability scale, through which 56 participants were excluded. All participants in the study were informed in advance, giving their written consent to participate in it voluntarily. Each participant was given the opportunity to carefully read the introductory informed consent before making their final decision. All participants had the right to ask questions and receive information related to the procedure and the opportunity to withdraw from the study at any time, regardless of the fact that the procedure did not pose any risks to the persons in it. All subjects were informed that no material and financial resources were provided for participation and that the information collected would be used in strict confidence only for the purposes of the study, which complied with all national and European General Data Protection Regulation (GDPR) rules.

The Connor-Davidson Resilience Scale (CD-RISC) consists of 25 statements, each rated according to a 5-point scale (0-4) and higher scores reflect greater resilience [29]. According to the authors, mental resilience can be an assessment measure concerning the ability to cope with stress and, as such, is an important factor in reducing stress reactions, anxiety, and depression [28-30] (in the present study, Cronbach's alpha=0.89).

The Perceived Stress Scale (PSS) [24] is one of the most popular tools for measuring psychological stress. This is a questionnaire made independently that measures the extent to which people assess situations and their lives as stressful [24]. The elements of PPS assess the extent to which people believe they lived unpredictably, uncontrollably, and overwhelmed during the previous month [24]. The evaluated elements are more general in nature than focusing on specific events or experiences [26] (in the present study, Cronbach's alpha=0.86).

The Emotional Regulation Assessment Questionnaire (ERQ-1) is designed to assess individual differences using two emotional regulation strategies: cognitive reappraisal and expressive suppression. It is associated with statements about the assessment of self-control, regulation, and management of emotions. The statements relate to two aspects: emotional experience and emotional expression [27] (in the present study, Cronbach's alpha=0.81).

For the purpose of the study, filling out the Bus-Perry Aggression Questionnaire (AQ) [23] validated methodologies adapted for Bulgaria. The AQ is a 29-item instrument designed by Buss and Perry. This instrument evaluates several components of the construct: anger, verbal aggression, physical aggression, and hostility, and it has shown adequate psychometric standards in English-speaking samples [23] (in the present study, Cronbach's alpha=0.87).

Ethical procedure

The present study was approved by the Committee on Scientific Ethics at the Medical University of Plovdiv with number 3229 and protocol number 7 of 17.11.2021, with the justification that the scientific research meets the standards and criteria for scientificity and ethics, consistent with the requirements of the Declaration of Helsinki for ethics in science.

Statistical methods

For the purposes of the research, the following statistical methods were used: alternative analysis, variance analysis, and analysis of variance (ANOVA). ANOVA was employed to test hypotheses for equality between more than two means. The differences were considered statistically significant when they exceeded a critical value of u for α=0.05. The IBM SPSS Statistics for Windows, Version 21 (Released 2012; IBM Corp., Armonk, New York, USA) was used for the computer processing of the collected database. Regression analysis was conducted to examine the associations between psychological factors and HbA1c levels. Confounding variables such as age, gender, duration of diabetes, weight, and physical activity were controlled for in the model to ensure that the relationships observed were not influenced by these factors.

## Results

The total number of participants was 126, aged between 45 and 72 years (SD: 8.969±0.799); of which 84 people were diagnosed with T2DM, divided into two groups: 42 people with poor glycemic control (HbA1c>7.5%) and 42 people with good and satisfactory glycemic control (HbA1c<7.5%). The third group is a healthy control one, consisting of 42 people in the same age group who have no chronic diseases. The distribution by gender is 65 (51.6%) men and 61 (48.4%) women. Of these, 42 people were patients with T2DM with poor glycemic control (HbA1c>7.5%) (32.8% women and 33.8% men), 42 people were patients with T2DM with good and satisfactory glycemic control (HbA1c<7.5%) (34.4% women and 32.3% men), and 42 people without established chronic diseases (32.8% women and 33.8% men).

The three groups have been made uniform in terms of the main demographic characteristics: gender (p>0.05; χ^2^=0.967) and age (p>0.05; F=0.314). The distribution by factor variables and glycosylated hemoglobin levels is presented in Table 1.

**Table 1:** Distribution by glycosylated hemoglobin levels (HbA1c>7.5%, HbA1c<7.5%, and healthy control group) and variables. Std. Er.: standard error

	HbA1c>7.5%		HbA1c<7.5%		Healthy control group	
Variable	Mean or %	SD±Std. Er.	Mean or %	SD±Std. Er.	Mean or %	SD±Std. Er.
Age	57.64	9.842±1.52	57.26	10.095±1.56	58.76	6.727±1.04
Weight (kg)	101.55	23.006±3.55	94.93	20.172±3.11	83.36	14.593±2.25
Height (cm)	171.07	10.868±1.68	167.52	10.287±1.59	164.60	24.908±3.84
Physical activity (hours per week)	21.31	23.676±3.65	25.21	34.655±5.35	29.26	32.018±4.85
Cigarettes (% yes)	33.30		45.20		40.50	
Coffee (% yes)	85.70		78.60		64.30	
Alcohol (% yes)	88.10		61.90		59.50	
Sleep disorders (% yes)	33.30		33.30		21.40	

The analysis by ANOVA (Table 2) shows that the comparison between the studied groups on HbA1c and the individual psychological characteristics has statistically significant differences in resilience (F=9.136; p<0.001), perceived stress (F=30.582; p<0.001), emotional regulation-suppression (F=4.186; p<0.05), and expression of anger (F=4.294; p<0.05).

**Table 2:** Comparison of the studied groups (poor HbA1c control, good HbA1c control, and healthy control) by mean values (mean) relative to individual psychological characteristics. PSS: Perceived Stress Scale; CD-RISC: Connor-Davidson Resilience Scale; FA: physical aggression; VA: verbal aggression; ERQ: emotional regulation; Ang: anger; Hos: hostility

Scales	Groups	N	Mean	SD	F	p-value
CD-RISC	Healthy control	42	79.69	12.633	9.136	<0.001
	HbA1c<7.5%	42	75.62	12.593		
	HbA1c>7.5%	42	70.98	12.420		
PSS	Healthy control	42	37.14	9.348	30.582	<0.001
	HbA1c<7.5%	42	37.26	7.840		
	HbA1c>7.5%	42	48.52	5.214		
ERQ_ REAS	Healthy control	42	31.36	8.006	1.428	>0.05
	HbA1c<7.5%	42	29.19	7.696		
	HbA1c>7.5%	42	28.64	7.644		
ERQ_ SUP	Healthy control	42	17.52	6.794	4.186	<0.05
	HbA1c<7.5%	42	17.52	6.794		
	HbA1c>7.5%	42	19.31	5.491		
Ang	Healthy control	42	19.19	5.602	4.294	<0.05
	HbA1c<7.5%	42	18.05	6.262		
	HbA1c>7.5%	42	21.62	5.198		
FA	Healthy control	42	19.19	5.602	1.824	>0.05
	HbA1c<7.5%	42	18.05	6.262		
	HbA1c>7.5%	42	21.62	5.198		
VA	Healthy control	42	20.00	6.648	0.286	>0.05
	HbA1c<7.5%	42	20.88	6.605		
	HbA1c>7.5%	42	22.81	7.402		
Hos	Healthy control	42	16.00	3.663	1.476	>0.05
	HbA1c<7.5%	42	16.00	3.414		
	HbA1c>7.5%	42	16.48	2.865		

The multiple comparisons analysis shows that there is a statistical difference between the groups of subjects in the juxtaposition of mental resilience, perceived stress, suppression of emotional regulation, and anger on the scales of aggression (p<0.05). At the same time, no significant difference was found in the cognitive reappraisal of emotional regulation, as well as in the other scales of aggression (physical and verbal aggression, hostility, and general aggression) between the different groups (p>0.05).

When comparing the mean values of mental resilience with the levels of glycosylated hemoglobin, we find that there are statistically significant higher mean values between the groups HbA1c>7.5% and the healthy control (p>0.05 for good HbA1c control, p<0.01 for healthy control). This result is also confirmed by comparing the mean values of perceived stress with the levels of glycosylated hemoglobin, as we find that there are statistically higher mean values between the group with poor glycemic control HbA1c>7.5%, comparable to the group HbA1c<7.5% and the healthy group (p<0.05 for HbA1c<7.5%, p<0.05 for healthy control). A statistically significant difference was also found when comparing the mean values of the scale of suppression of emotional regulation with the levels of glycosylated hemoglobin, with statistically higher mean values between the group with poor glycemic control, comparable with the healthy group (p>0.05 for HbA1c<7.5%, p<0.05 for healthy control). Interesting is the result when comparing the average values of anger with the levels of glycosylated hemoglobin, where we find that there is a statistical difference between the group with poor glycemic control, comparable to the group with HbA1c<7.5% (p<0.05 for HbA1c<7.5%, p>0.05 for healthy control).

Linear regression

In the first stage of the regression analysis, socio-demographic variables such as gender and age were included in the model. The initial analysis identified no significant correlations between these variables and the dependent outcomes, indicating their weak influence on the overall predictive model (β=-0.034, p=0.784 for gender; β=0.045, p=0.672 for age). Although these variables were included in the analysis, their minimal impact led to a focus on stronger predictors in subsequent stages of the regression.

In the second stage, we compared the individual factorial and resultant predictors with the levels of glycosylated hemoglobin. Each predictor was tested one at a time, separately in a model (Table 3). This stage helped us to specify the more significant predictors that make up the current regression model.

**Table 3:** Connection between the different factors and the three groups of HbA1c (poor glycemic control, good glycemic control, and healthy control group). PSS: Perceived Stress Scale; CD-RISC: Connor-Davidson Resilience Scale; GA: general aggression; ERQ: emotional regulation *p<0.05; **p<0.01; ***p<0.001

Factors	Unst. β	Stand. β	R²	F	Sig.
CD-RISC	-0.601	-0.359***	0.129	18.334	0.000
PSS	0.044	0.502***	0.252	41.708	0.000
ERQ-R	-0.015	-0.142	0.020	2.568	0.112
ERQ-S	0.032	0.226*	0.051	6.665	0.011
Anger	0.024	0.170*	0.029	4.694	0.047
GA	0.008	0.151*	0.023	4.881	0.042
Weight	0.016	0.505***	0.249	42.432	0.000
Physical activity	-0.007	-0.223*	0.050	6.465	0.012
Alcohol intake	0.129	0.263**	0.062	9.206	0.003
Smoking	-0.006	-0.048	-0.006	.286	0.594
Coffee intake	-0.030	-0.046	0.002	.262	0.610
Sleep problem	0.224	0.137	0.019	2.357	0.127

From the factor structure (Table 3), we conclude that the greatest predictive results are the psychological characteristics, positively related to perceived stress (β=0.502; p<0.001) and vice versa to mental resilience (β=-0.359; p<0.001). Less influential is the direct dependence on emotional regulation-expressive suppression (β = 0.226; p<0.05), the positive correlation with anger (β=0.170; p<0.001), and general aggression (β=0.151; p<0.05). We found a good positive correlation with the factorial factor "weight" (β=0.505; p<0.001), a moderate correlation with alcohol intake (β=0.263; p<0.01), and feedback on physical activity (β=-0.223; p<0.05).

Comparing the two separate groups with good and poor glycated hemoglobin control to determine whether the group moderated the effect of the predictors, it was found that the group with poor glycemic control had the highest level of influence on the overall model, affecting approximately 80% of the studied predictors (R^2^=0.812; p<0.001). From the performed collinearity diagnostics, we note acceptable levels of correlation and multicollinearity, with tolerance coefficients varying between 0.903 and 0.780 and for VIF between 1.790 and 1.107. Over 67% of the studied predictors were predictive for both groups (R^2^=0.675; p<0.001; df=83).

In the third stage, after determining the significant variables, we applied the step-by-step method of regression analysis to verify the overall model. From the performed collinearity diagnostics, we note acceptable levels of correlation and multicollinearity, with tolerance coefficients varying between 0.916 and 0.821 for VIF between 1.835 and 1.002 (Table 4).

**Table 4:** Factor model of the three HbA1c groups (poor glycemic control, good glycemic control, and healthy control). *p<0.05; **p<0.01; ***p<0.001 PSS: Perceived Stress Scale; CD-RISC: Connor-Davidson Resilience Scale; VIF: variance inflation factor

Dep. variable	Predictors		β	R^2^	ΔR^2^	F	p	Collinearity	
								Tolerance	VIF
HbA1c	Step 1:	Weight	0.505***	0.255	0.249	42.432	0.000	-	-
	Step 2:	Weight	0.370***						
		PSS	0.365***	0.370	0.359	36.059	0.000	0.863	1.159
	Step 3:	Weight	0.360***					0.861	1.161
		PSS	0.307***					0.862	1.159
		CD-RISC	-0.225**	0.416	0.402	28.990	0.000	0.914	1.002
	Step 4:	Weight	0.333***					0.836	1.176
		PSS	0.311***					0.862	1.159
		CD-RISC	-0.206**					0.914	1.006
		Alcohol	0.160*	0.438	0.420	23.623	0.000	0.916	1.038
	Step 5:	Weight	.325***					0.821	1.835
		PSS	0.322***					0.862	1.159
		CD-RISC	-0.160**					0.914	1.006
		Alcohol	0.169*					0.916	1.038
		Physical activity	-0.169*	0.465	0.443	20.856	0.000	0.846	1.241

When performing the stepwise method of linear regression (Table 4), a valid model was identified, consisting of five steps and five predictors, two of which are related to psychological characteristics, namely perceived stress and resilience, and three socio-demographic predictors: weight, alcohol intake, and physical activity. By regression analysis, for step one, the most significant predictor is weight with a predictive effect of 25% (R^2^=0.255; p<0.001). In the second step, the predictor perceived stress was added with an increased predictive effect of 37% (R^2^=0.370; p<0.001) for both factors. The third step is related to the appearance of resilience and increased predictability up to 44% (R^2^=0.370; p<0.001). The fourth and fifth steps, related to the addition of two socio-demographic predictors, alcohol intake and physical activity, complete the final model with 46% (R^2^=0.465; p<0.001) cause-and-effect impact on the subjects (Table 4).

## Discussion

The recognition of diabetes mellitus, particularly T2DM, which represents about 90% of all diabetes cases, as a global health crisis with substantial social implications has led to a consensus on the need for a comprehensive approach to its management. Traditionally, diabetes research and treatment have focused predominantly on the somatic or physical aspects of the disease. However, there is a growing understanding that to effectively address this pervasive health issue, a broader perspective that includes psychological factors is essential. This shift toward a more integrated approach is driven by the acknowledgment that the onset and progression of T2DM are influenced not only by physical but also by psychological components, such as personality traits, cognitive processes, emotional states, and behavioral characteristics [1,3,5]. It is difficult to determine the accuracy of the many psychological factors influencing the course of the disease in people with chronic diseases, in particular those suffering from T2DM, or on the change in the quality of life of these patients [6]. Psychological factors are part of the predictors that are associated with changes in reactions in the process of adaptation in individuals with T2DM. Due to the presence of many factors, socio-demographic, psychological, and physiological, it is difficult to determine the exact etiological relationship in people with diabetes. Despite this recognition, research into the connection between psychological factors and T2DM is relatively sparse. This gap in research underscores the necessity of adopting a multidisciplinary approach that encompasses both somatic and psychological predictors to enhance our understanding and management of diabetes. Existing studies in this field have begun to uncover distinct psychological profiles among individuals with T2DM, identifying prevalent characteristics such as hostility, anger, ambition, emotional instability, social isolation, negative emotions, neuroticism, and psychoticism. These findings suggest a complex interplay between psychological well-being and the physical manifestation of diabetes, indicating that individuals with T2DM may be predisposed to specific emotional and behavioral patterns. Whether a chronic condition changes the psychological structure of the personality (psychopathologically) or psychological characteristics are a real predictor of the onset and development of the disease are questions that require numerous cross-cultural studies [4,17].

The study of the connection between resilience and stress on HbA1c levels is poorly represented.

One study examined whether levels of resistance protect patients with diabetes from worsening HbA1c during increasing distress [31]. Other research in the field has found a link between maladaptive strategies and poor glycemic control on the one hand and quality of life and psychological adaptation on the other [19,32].

ANOVA analysis revealed statistical differences for the psychological predictors relative to the group of individuals with poor glycated hemoglobin control and the other two groups. The largest difference was found for resilience between the HbA1c-poor control group and the healthy control. In perceived stress between the poor HbA1c control and good HbA1c control group. And to a lesser extent as a predictor of emotional regulation-suppression between the poor HbA1c control and the healthy group. For the predictor of emotional expression through anger, the statistical difference was between poor HbA1c control and good HbA1c control.

From the present study, we can conclude that we note that the level of glycosylated hemoglobin in patients with poor glycemic control, good and satisfactory control, and a healthy control group is influenced by the presence of two more pronounced psychological predictors, namely the subjective perception of stressful situations and the presence or absence of resilience. Studies aimed at establishing a link between resilience and HbA1c did not find statistically significant differences in this characteristic [17], but in one of them, it was found over time that resilience predicted a change in glycemic control [18].

There are other psychological characteristics in the present study that are less well represented as predictors, such as emotional regulation concerning the suppression of the present state, as well as the manifestation of anger and general aggression (including verbal and physical aggression, hostility). These results are confirmed by previous studies, which found that anger and emotional suppression of aggressive emotions have a direct correlation with higher levels of HbA1c [17,18,32].

From socio-demographic predictors, we find a significant influence of weight, alcohol use, and the presence or absence of physical activity. Combining the effective with the factorial predictors, we find a predictive pattern in 46% of these predictors, relative to the levels of glycosylated hemoglobin in the subjects.

Research on psychological characteristics such as resilience, emotional regulation, and aggression as predictors of the onset and development of diabetes mellitus type 2 is still an insufficiently well-established cause-and-effect connection. As the current study is cross-sectional, more in-depth and long-term research is needed in this area to confirm resilience, perceived stress, and emotional regulation as predominant psychological predictors of HbA1c levels. The implications of these findings are profound for clinical and health psychology, highlighting the importance of incorporating psychological assessment and intervention into the comprehensive care of patients with T2DM. By addressing the psychological aspects of diabetes alongside the physical, healthcare professionals can offer a more holistic treatment approach. This not only acknowledges the multifaceted nature of diabetes but also opens up avenues for potentially more effective management strategies that consider the patient's mental well-being as integral to their overall health. This study emphasizes a multidisciplinary approach that integrates psychological characteristics such as resilience, perceived stress, and emotion regulation with glycemic control in patients with type 2 diabetes. This link between mental health and physiological outcomes offers a holistic view of psychosomatic factors and diabetes 2 management.

In summary, the integration of psychological factors into the understanding and treatment of T2DM represents a pivotal shift toward a more holistic approach to healthcare. This perspective not only enhances our comprehension of diabetes but also offers a more nuanced and effective approach to its management. As research in this area continues to evolve, it holds the promise of leading to improved outcomes for patients by addressing the intricate relationship between mind and body in the context of diabetes care.

Limitations of the study

This study has several limitations that should be considered when interpreting the results. The cross-sectional design provides only a snapshot of the associations between psychological characteristics and HbA1c levels in patients with type 2 diabetes. Therefore, definitive conclusions regarding cause-and-effect relationships cannot be made, and longitudinal follow-up studies are necessary. The study included a limited set of psychological factors, such as resilience, perceived stress, emotion suppression, aggression, and anger, which narrows the scope of the analysis. Other factors, such as depression and social support, which may influence diabetes control, were not considered. Although factors such as weight, physical activity, and alcohol consumption were accounted for, the lack of control over other variables limits the ability to identify the full spectrum of influences on HbA1c levels. Additionally, the geographical scope of the study was limited to a single hospital in Bulgaria, making it difficult to generalize the findings to other regions or cultural contexts. These limitations highlight the need for more long-term, large-scale, and multifactorial studies to expand upon the current findings and provide a better understanding of the relationship between psychological factors and glycemic control in type 2 diabetes.

The study has several limitations that should be acknowledged. The relatively small sample size of 84 patients may limit the generalizability of results to larger and more diverse populations of individuals with type 2 diabetes. In addition, the cross-sectional study design collected data at only one point in time, hindering the ability to establish causal relationships between psychological factors and HbA1c levels. Future longitudinal studies are needed to determine the direction of these associations. Finally, potential confounders such as medication use, lifestyle habits (e.g., diet and exercise), and socioeconomic status were not accounted for in the analysis. These factors could influence the observed associations and affect the reliability of the study findings.

## Conclusions

The present study focuses on the relationship of psychological factors in patients with type 2 diabetes, which have been understudied in the current scientific literature. The statistical difference in the factors, resilience, PSS, and suppression of emotion regulation, was highlighted in the individuals studied for the respective groups. A statistical difference was found between the HbA1c poor control group and the healthy group on resilience and PSS. From the factor structure, we conclude that the psychological characteristics positively associated with perceived stress (β=0.502; p<0.001), inversely associated with resilience (β=-0.359; p<0.001), emotion regulation-expressive suppression (β=0.226; p<0.05), positively correlated with anger (β=0.170; p<0.001), and general aggressiveness (β=0.151; p<0.05) were predictive. Linear regression established a valid model consisting of five predictors, psychological characteristics (stress and resilience), and three socio-demographic predictors (weight, alcohol intake, and physical activity), with 46% (R²=0.465; p<0.001) causal influence for the subjects.
